# The relationship between contributions of authors and author order

**DOI:** 10.1002/jgf2.466

**Published:** 2021-06-01

**Authors:** Rieko Ueda, Yuji Nishizaki, Yasuhiro Homma, Patrick Devos, Shoji Sanada

**Affiliations:** ^1^ Medical Technology Innovation Center Juntendo University Tokyo Japan; ^2^ Department of Cardiovascular Biology and Medicine Graduate School of Medicine Juntendo University Tokyo Japan; ^3^ Department of Orthopedic Surgery Juntendo University School of Medicine Tokyo Japan; ^4^ Univ Lille CHU Lille, Lillometrics Lille France; ^5^ Clinical and Translational Research Center Kobe University Hospital Kobe Japan

## CONFLICT OF INTEREST

The authors have stated explicitly that there are no conflicts of interest in connection with this article.

To the Editor,

Research achievements are often evaluated on the basis of the impact factor of journals and the level of contribution by each author. In biomedical fields, individual research contribution is usually reflected by the author order in publications. In France, the System for the Identification, Management and Analysis of Scientific Publications (SIGAPS) score is used to evaluate the quality of papers, including the value of the author order.^1^, ^2^ However, the weight of the author order could differ by country. We, therefore, compared the value of the author order in France and Japan in accordance with questionnaire surveys in our previous research.^3^ Our previous findings revealed that, apart from the first author, in France, the last author is highly esteemed, in contrast to Japan, where the second author is highly esteemed.^3^ We evaluated the relationship between the author order and their research contributions from a novel perspective in comparison with our earlier research.^3^

The corresponding author is considered to have made the greatest research contribution and played the most important role in the process from writing the paper to the submission and in responding to peer reviews. Therefore, we extracted 580 clinical research papers from business reports pertaining to core clinical research hospitals that were submitted in 2017 and published online by the Ministry of Health, Labour and Welfare.^4^ We then summarized the position in which the corresponding author was most frequently listed in the actual paper: first, second, third, penultimate or last, or in some other position.

We examined a total of 576 papers, excluding four with more than one corresponding author. As shown in Table 1, the majority (54%) of corresponding authors were first authors, fewer were second authors (24%) or last authors (19%), and only 1% each were third authors, penultimate authors, or in some other position.

**TABLE 1:** Position of corresponding authors in 576 academic papers

First author	312 (54%)
Second author	138 (24%)
Last author	107 (19%)
Third author	8 (1%)
Penultimate author	8 (1%)
Other contributing authors	3 (1%)

With regard to the value of author order in papers in Japan, first and second authors are at a higher level than the last author, which differs from the usual way of thinking in France. This may be because, in Japan, regardless of the degree of practical involvement in research, the supervisor of the study, who often plays a key role in securing funding and as the public “face” of the research, tends to be listed as the last author.^3^

The position of the penultimate author is more highly esteemed in France than in Japan.^3^ This may reflect differences in joint research activities. In France, much research is multidisciplinary and involves several research units; therefore, the contribution of each researcher and unit is highly valued. This could partly explain the differences in the author order observed between France and Japan.

In this study, we investigated the relationship between the contributions of authors and author order only in France and Japan. We next plan to collect and evaluate similar data from other countries as well.
